# Dynamic parameter identification and adaptive control with trajectory scaling for robot-environment interaction

**DOI:** 10.1371/journal.pone.0287484

**Published:** 2023-07-13

**Authors:** Ke Song, Heyu Hu

**Affiliations:** 1 Electronic Engineering Institute, Xi’an Aeronautical University, Xi’an, China; 2 Zhongyuan University of Technology, Zhengzhou, China; Hebei University of Technology, CHINA

## Abstract

To improve the force/position control performance of robots in contact with the environment, this paper proposes a control scheme comprising dynamic parameter identification, trajectory scaling, and computed-torque control based on adaptive parameter estimation. Based on the Newton–Euler method, the dynamic equation and its regression matrix is obtained, which is helpful to reduce the order of the model. Subsequently, the least-square method is implemented to calculate the values of the basic parameters of the dynamics. The identified dynamic parameters are used as initial values in the adaptive parameter estimation to obtain the torque, and trajectory scaling is applied to control the contact force between the robot and the environment. Finally, the dynamic parameter identification method and control algorithm are verified by conducting a simulation. The results show that the comprehensive application can help improve the control performance of robots.

## 1 Introduction

Robots are being increasingly deployed for modern industrial production. Engineers and designers have developed a wide variety of robots for various application scenarios and task requirements [1]. Given that a robot is a typical nonlinear and strongly coupled electromechanical system, the control performance has been the focus in robotics research. To improve the control accuracy of robots, the impedance control and hybrid position/force control [2, 3], have been developed and widely used.

An accurate dynamic model is the basis for robot control, while an inaccurate model can affect the control performance and even lead to instability [4]. Although the inertial parameters of a robot take certain constant values, some of these parameters are unknown even to the robot manufacturer because of the complexity of the robot components and assembly errors. Because of the nonlinear and strong coupling characteristics of robots, researchers have used intelligent control methods to approximate the dynamic model, such as the neural network system [5–7] and reinforcement learning method [8]. However, these methods can only prove the consistency and boundedness of the tracking error and cannot ensure that the estimated value converges to the real value [9–11]. The computational efficiency of robot dynamics models established by forward- and backward-iteration Newton–Euler method has been proven to be sufficient.

Accurate dynamic parameters cannot be obtained in advance, which is an obstacle that must be overcome to establish a dynamic model [12, 13]. To this end, researchers have developed a method for dynamic parameter identification. In [14], the spinor method combined with the recursive Newton–Euler method is used to derive an analytical dynamic expression; however, the column vectors of the regression matrix given were linearly dependent. In [15, 16], a new method is provided to obtain the maximum linearly independent group of the column vectors of a regression matrix. Thereafter, the least-squares method is applied to find the parameter matrix. To identify the dynamic parameters easily, it is necessary to find the excitation signal, so as to minimize the conditional number of the observation matrix [17, 18]. [19] introduces the application of QR decomposition and SVD decomposition to extract the regression matrix and dimensionality reduction, so as to obtain the minimum parameter set. [20] implements the least-squares method to identify the unknown inertial parameters, and [21–23] introduce the application of a recursive least-squares method. In recent years, artificial intelligence methods have been successfully applied in many fields, including for determining the dynamic parameters associated with robots. The model identification method proposed in this paper is based on the Newton–Euler principle, with advantages such as improved identification process, reduced computational complexity, and ease of use.

The control of the contact force is another important task that needs to be solved. Force control is typically realized using two methods: hybrid force/position control and impedance control [24, 25]. For the former, position and force control loops are designed, with the two loops being relatively independent. Based on the interaction between the robot and the environment, the impedance control method realizes the force position control of the manipulator by establishing an impedance model between the position and the force. Unlike the above two methods, trajectory scaling involves incorporating the force error into the position correction to achieve position control [26–28]. Compared with the impedance control, the trajectory scaling method changes the trajectory planning of the timestamp, i.e., the revised one tracks the desired trajectory well before. Compared with the hybrid control method for the force position, the trajectory scaling method is implemented only after the robot contacts the environment. Before contacting, the robot is in the position control mode. After the contact, the force position control will be carried out, without switching between the two states, and the control process will be more stable. Control precision is one of the important indexes in the design of controllers, and the precision of the control algorithm directly depends on the modeling precision of the dynamic model. [29] combined iterative learning and reinforcement learning methods to improve the force tracking accuracy. In [30, 31], an adaptive control algorithm is designed based on the computed-torque algorithm and the method of adaptive dynamic parameter estimation in [32, 33], where a high-precision dynamic control is realized.

Currently, the idea of double-loop control has been widely adopted, i.e., force control for the outer loop and position control for the inner loop [34, 35]. In this study, a trajectory scaling method is used to control the outer loop. The basic idea is to change the desired trajectory by obtaining the error between the actual joint torque and the desired joint torque, so as to indirectly realize the expected contact force control. Based on dynamic parameter identification, an adaptive control scheme is introduced to improve the control precision. The main contributions of this paper are as follows:

1. To identify the dynamic parameters of the robot, the dimension of the regression matrix is reduced by QR and SVD decomposition to obtain the minimum set of dynamic parameters. Based on the collected values of the angle, angular velocity, angular acceleration, and joint torque, the dynamic parameters are calculated using the least-squares method, which is clear and simple.
2. To control the contact force, the idea of trajectory scaling is applied to the outer loop to make the end-effector of the robot follow the desired trajectory as far as possible. It is noted that the desired trajectory is varied by changing the timestamp in the trajectory generation process based on the torque error, which is helpful to indirectly realize the control of the contact force.
3. An adaptive control strategy based on the computed-torque method is adopted in the inner loop, so that the robot can adjust the dynamic parameters depending on the task and improve the control performance.

The rest of this paper is organized as follows. In Section 2, the overall scheme is introduced, including the dynamic parameter identification and control strategy design. In Section 3, we introduce the robot model and dynamic parameter identification method. In Section 4, we introduce the force control algorithms based on trajectory scaling and adaptive computation. Section 5 presents the simulation design and results. Section 6 concludes the paper.

## 2 Problem formulation

**Fig 1:**
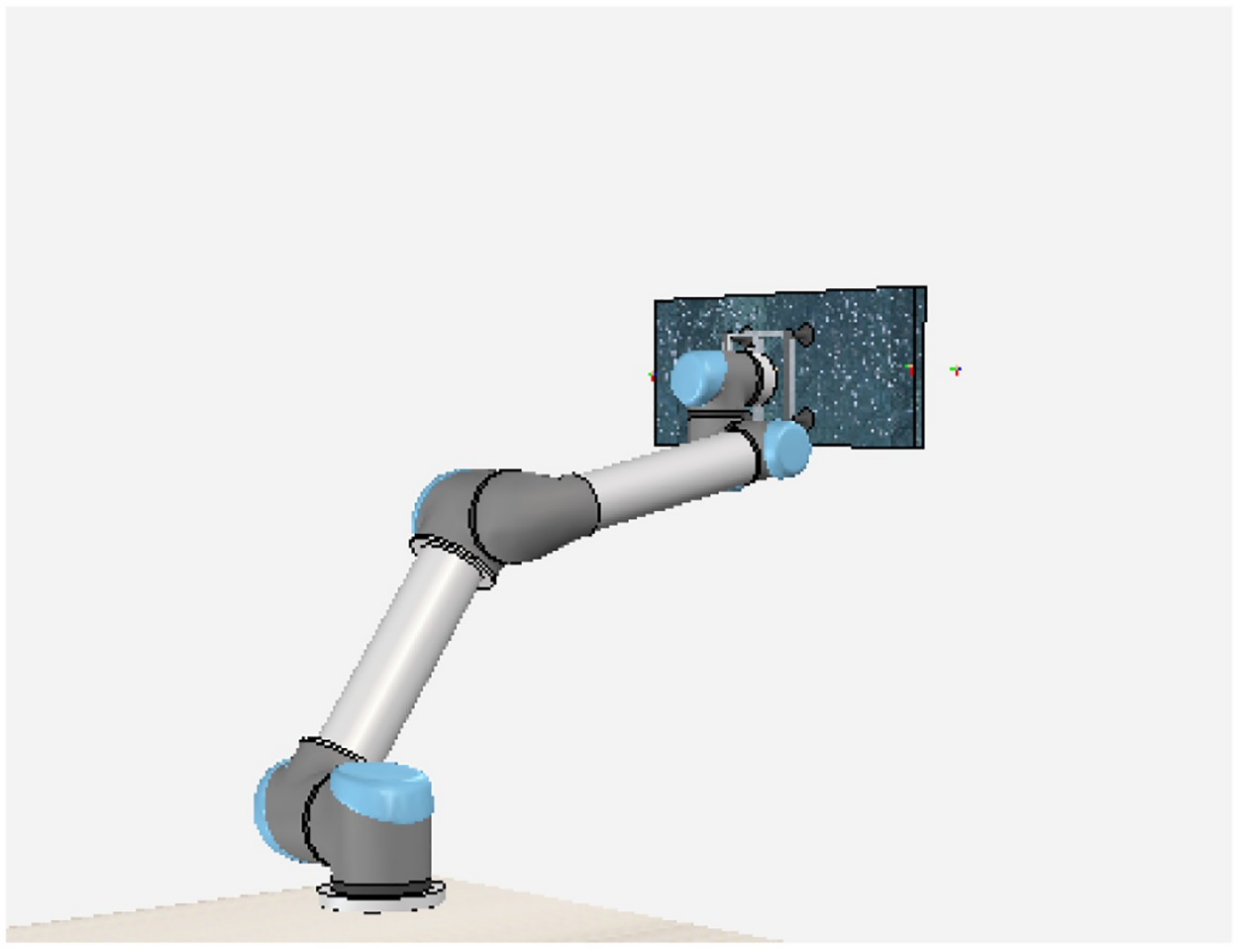
Schematic diagram of robot and environment.

Robot grasping objects moving in tash space is a typical working scenario. Taking the widely used UR5 robot as an example, we establish the model shown in Fig 1.

**Fig 2:**
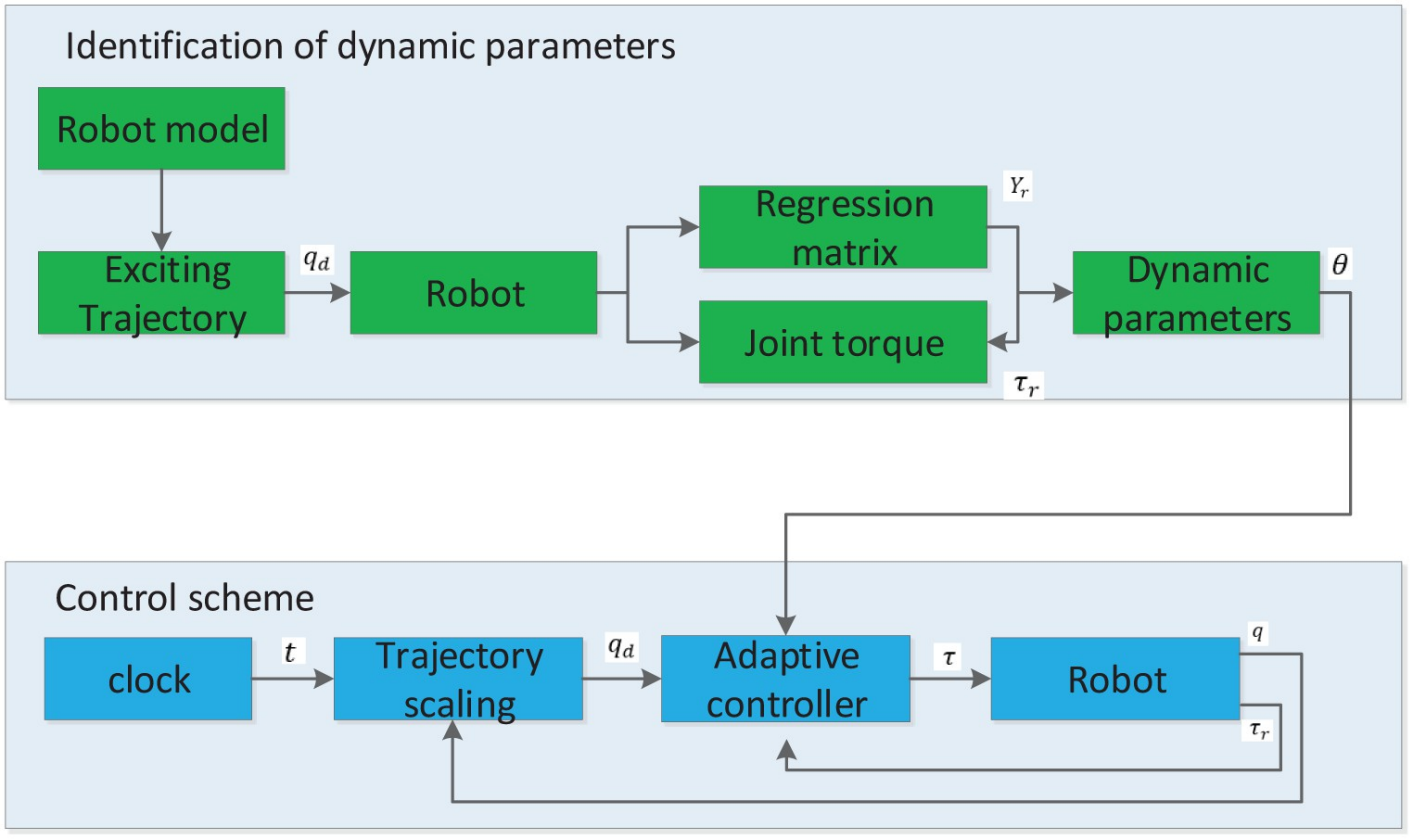
Parameter identification and control algorithm.

The structure of the parameter identification and control algorithm is shown as Fig 2, which consists of two parts. The objective of parameter identification is to obtain the inertial parameters related to the dynamics. Based on the identified dynamic parameters, the control algorithm controls the trajectory and contact force of the end-effector of the robot.

In the dynamic parameter identification module, an optimal excitation trajectory is generated based on the dynamic and kinematic models of the robot, and then the robot follows this trajectory. The joint angle and torque during this motion are collected, and the basic dynamic parameters are obtained based on the reduced-order regression matrix and least-squares algorithm. In the control scheme module, the outer loop is composed of trajectory scaling, and the inner loop is composed of an adaptive parameter estimation algorithm based on the computed torque. The objective of the former is to generate the desired trajectory with respect to the trajectory generator and the actual torque, so that the robot follows the planned trajectory as much as possible, and use this generated trajectory for the compliance of the contact force. The objective of the latter is to obtain the required torque of the robot and reduce the complexity of the algorithm using the dynamic formula based on the Newton–Euler method, in order to enable a precise control of the robot.

## 3 System model and dynamic parameter identification

### 3.1 System model

The kinematics model of the robot is established with respect to the D-H law. After establishing the coordinate system of each link using the preposition coordinate method, we determine the transformation relationship between the *i* − 1 coordinate system and the *i* coordinate system via the translation and rotation of each coordinate system [4]. The transformation process can be used to represent the homogeneous transformation matrix ${}_{i}^{i-1}R$, which can be expressed as

$$\begin{array}{c} {}_{i}^{i-1}R=[\begin{array}{cccc} \text{cos}\;q_{i} & -\;\text{sin}\;q_{i} & 0 & a_{i-1}\\ \text{sin}\;q_{i}\;\text{cos}\;\alpha_{i-1} & \text{cos}\;q_{i}\;\text{cos}\;\alpha_{i-1} & -\;\text{sin}\;\alpha_{i-1} & -d_{i}\;sin\;\alpha_{i-1}\\ \text{sin}\;q_{i}\;\text{sin}\;\alpha_{i-1} & \text{cos}\;q_{i}\;sin\;\alpha_{i-1} & \text{cos}\;\alpha_{i-1} & d_{i}\;\text{cos}\;\alpha_{i-1}\\ 0 & 0 & 0 & 1 \end{array}] \end{array}$$ (1)

where *a*_*i*_ is the length of the link, *α*_*i*_ is the twist angle of the link, *d*_*i*_ is the distance of the link, and *q*_*i*_ is the angle between the two links.

Lagrangian and Newton–Euler dynamics methods are typically used for robot dynamic models. For the former, the dynamic equation of the joint space is

$$\begin{array}{c} D\left(q\right)\ddot{q}+C\left(q,\dot{q}\right)+G\left(q\right)=\tau \end{array}$$ (2)

where *D*(*q*) is the inertia matrix, $C(q,\dot{q})$ is the centrifugal force and Coriolis force vector, and *G*(*q*) is the gravity vector. An important property of this model is its linearity, which can be expressed as

$$\begin{array}{c} D\left(q\right)\ddot{q}+C\left(q,\dot{q}\right)+G\left(q\right)=Y\left(q,\dot{q},\ddot{q}\right)P \end{array}$$ (3)

where $Y(q,\dot{q},\ddot{q})$ represents the regression matrix of the dynamics, and *P* represents the inertial parameter matrix. This property is the basis of the robot dynamic parameter identification and control. The Newton–Euler equation is used to derive the inverse dynamics calculation formula, which includes forward and backward processing.

Forward:

$$\begin{array}{c} {\tilde{\omega }}_{i}={}_{i-1}^{i}R\left({\tilde{\omega }}_{i-1}+z{\dot{q}}_{i}\right)\left({\tilde{\omega }}_{0}=0\right) \end{array}$$ (4)

$$\begin{array}{c} {\tilde{\varepsilon }}_{i}={}_{i-1}^{i}R\left[{\tilde{\varepsilon }}_{i-1}+{\tilde{\omega }}_{i-1}\times z{\dot{q}}_{i}+z{\ddot{q}}_{i}\right]\left({\tilde{\varepsilon }}_{0}=0\right) \end{array}$$ (5)

$$\begin{array}{c} {\tilde{v}}_{i}={}_{i-1}^{i}R\left({\tilde{v}}_{i-1}+z{\dot{q}}_{i}\right)+{\tilde{\omega }}_{i}\times {\tilde{p}}_{i}^{*} \end{array}$$ (6)

$$\begin{array}{c} {\tilde{a}}_{i}={}_{i-1}^{i}R\left({\tilde{a}}_{i-1}+z{\ddot{q}}_{i}\right)+{\tilde{\varepsilon }}_{i}\times {\tilde{p}}_{i}^{*}+{\tilde{\omega }}_{i}\times \left({\tilde{\omega }}_{i}\times {\tilde{p}}_{i}^{*}\right) \end{array}$$ (7)

$$\begin{array}{c} {\tilde{a}}_{Ci}={\tilde{a}}_{i}+{\tilde{\varepsilon }}_{i}\times {\tilde{r}}_{Ci}+{\tilde{\omega }}_{i}\times \left({\tilde{\omega }}_{i}\times {\tilde{r}}_{Ci}\right) \end{array}$$ (8)

$$\begin{array}{c} {\tilde{F}}_{i}=m_{i}{\tilde{a}}_{Ci} \end{array}$$ (9)

$$\begin{array}{c} {\tilde{N}}_{i}={\tilde{I}}_{Ci}{\tilde{\varepsilon }}_{i}+{\tilde{\omega }}_{i}\times {\tilde{I}}_{Ci}{\tilde{\omega }}_{i} \end{array}$$ (10)

Backward:

$$\begin{array}{c} {\tilde{f}}_{i}={\tilde{F}}_{i}+{}_{i+1}^{i}R{\tilde{f}}_{i+1}\left({\tilde{f}}_{n+1}=0\right) \end{array}$$ (11)

$$\begin{array}{c} {\tilde{n}}_{i}={\tilde{N}}_{i}+{}_{i+1}^{i}R{\tilde{n}}_{i+1}+\left({\tilde{p}}_{i}^{*}+{\tilde{r}}_{Ci}\right)\times {\tilde{F}}_{i}+{\tilde{p}}_{i}^{*}\times \left({}_{i+1}^{i}R{\tilde{f}}_{i+1}\right)\left({\tilde{n}}_{i+1}=0\right) \end{array}$$ (12)

$$\begin{array}{c} \tau_{i}={\left({}_{i-1}^{i}Rz\right)}^{T}{\tilde{n}}_{i} \end{array}$$ (13)

where *z* is the unit vector in the Z axis; ${\tilde{\omega }}_{i}$ is the angular velocity; ${\tilde{v}}_{i}$ denotes the linear velocity; ${\tilde{\varepsilon }}_{i}$ denotes the angular acceleration; ${\tilde{a}}_{i}$ is the linear acceleration; ${\tilde{a}}_{Ci}$ is the linear acceleration of the center of mass; ${\tilde{I}}_{Ci}$ is the moment of inertia; ${\tilde{F}}_{i}$ is the inertial force; ${\tilde{N}}_{i}$ is the moment; ${\tilde{f}}_{i}$ is the joint force; ${\tilde{n}}_{i}$ is the joint torque; $\tau_{i}$ is the driving moment; ${\tilde{r}}_{Ci}$ is the centroid coordinate. All the above variables are defined in frame *i*. ${\tilde{p}}_{i}^{*}$ is the translation operator, ${\tilde{p}}_{i}^{*}=[\begin{array}{ccc} a_{i} & d_{i}\;\text{sin}\;\alpha_{i} & d_{i}\;\text{cos}\;\alpha_{i} \end{array}]$.

### 3.2 Identification of dynamic parameters

#### 3.2.1 Regression function

The robot dynamics equation established using the Newton–Euler method can be expressed using 4–11. However, it should be noted that the above formula describes the mass distribution of the link *i* using the mass *m*_*i*_ and the inertia tensor ${\tilde{I}}_{Ci}$ towards its center of mass, instead of using the product of the mass $m_{i}$, mass and the diameter of center of mass $m_{i}{\tilde{r}}_{Ci}$ and the inertia tensor matrix ${\tilde{I}}_{i}$ to describe the mass distribution of the link. Therefore, to simplify the robot dynamics calculation with the minimum inertial parameter, it is necessary to modify the relevant dynamics calculation formula so that it contains $m_{i}$, $m_{i}{\tilde{r}}_{Ci}$, and ${\tilde{I}}_{i}$. Since the dynamic characteristics of a robot are linear functions of its inertial parameters, the identification problem of the robot parameters can be transformed to a problem of solving linear equations. The dynamic Eqs 2 and 3 can be simplified as

$$\begin{array}{c} {\tilde{N}}_{i}^{*}={\tilde{N}}_{i}+\left({\tilde{p}}_{i}^{*}+{\tilde{r}}_{Ci}\right)\times {\tilde{F}}_{i} \end{array}$$ (14)

where *q*, $\dot{q}$, $\ddot{q}$, and *τ* are known. Since $Y(q,\dot{q},\ddot{q})$ is a known function independent of the inertial parameter *P*, the problem of identifying *P* is transformed to a problem of solving a system of linear Eq 14. Since 14 is a system of order 10*n* equations containing *n* unknowns, it is necessary to know at least *q*, $\dot{q}$, $\ddot{q}$, and *τ* among the 10 different unknowns to identify C using 14.

We define:

$$\begin{array}{c} \tau =Y(q,\dot{q},\ddot{q})P \end{array}$$ (15)

Let ${\tilde{\omega }}_{i}={\left[\begin{array}{ccc} {\tilde{\omega }}_{x}^{i} & {\tilde{\omega }}_{y}^{i} & {\tilde{\omega }}_{z}^{i} \end{array}\right]}^{T}$, then we can define:

$S\left({\tilde{\omega }}_{i}\right)=[\begin{array}{ccc} 0 & -{\tilde{\omega }}_{z}^{i} & {\tilde{\omega }}_{y}^{i}\\ {\tilde{\omega }}_{z}^{i} & 0 & -{\tilde{\omega }}_{x}^{i}\\ -{\tilde{\omega }}_{y}^{i} & {\tilde{\omega }}_{x}^{i} & 0 \end{array}]$ ,

$K\left({\tilde{\omega }}_{i}\right)=[\begin{array}{cccccc} {\tilde{\omega }}_{x}^{i} & {\tilde{\omega }}_{y}^{i} & {\tilde{\omega }}_{z}^{i} & 0 & 0 & 0\\ 0 & {\tilde{\omega }}_{x}^{i} & 0 & {\tilde{\omega }}_{y}^{i} & {\tilde{\omega }}_{z}^{i} & 0\\ 0 & 0 & {\tilde{\omega }}_{x}^{i} & 0 & {\tilde{\omega }}_{y}^{i} & {\tilde{\omega }}_{z}^{i} \end{array}]$

Based on the parallel axis theorem: ${\tilde{I}}_{i}={\tilde{I}}_{Ci}+m_{i}\left({\tilde{r}}_{Ci}^{T}{\tilde{r}}_{Ci}I-{\tilde{r}}_{Ci}{\tilde{r}}_{Ci}^{T}\right)$, 9 and 15 are combined as follows:

$$\begin{array}{c} {}[\begin{array}{c} {\tilde{F}}_{i}\\ {\tilde{N}}_{i}^{*} \end{array}]=FN*P \end{array}$$ (16)

where,

*FN*_11_ = 0

${FN}_{12}=S\left({\tilde{\varepsilon }}_{i}\right)+S\left({\tilde{\omega }}_{i}\right)S\left({\tilde{\omega }}_{i}\right)$

${{FN}_{13}=\tilde{a}}_{i}$

${FN}_{21}=K\left({\tilde{\varepsilon }}_{i}\right)+S\left({\tilde{\omega }}_{i}\right)S\left({\tilde{\omega }}_{i}\right)$

${FN}_{22}=S\left({\tilde{p}}_{i}^{*}\right)\left[S\left({\tilde{\varepsilon }}_{i}\right)+S\left({\tilde{\omega }}_{i}\right)S\left({\tilde{\omega }}_{i}\right)\right]-S\left({\tilde{a}}_{i}\right)$

${FN}_{23}=S\left({\tilde{p}}_{i}^{*}\right){\tilde{a}}_{i}$ .

We define the matrix *A*_*i*_, where:

*A*_*i*11_ = 0

$A_{i12}=S\left({\tilde{\varepsilon }}_{i}\right)+S\left({\tilde{\omega }}_{i}\right)S\left({\tilde{\omega }}_{i}\right)$

${A_{i13}=\tilde{a}}_{i}$

$A_{i21}=K\left({\tilde{\varepsilon }}_{i}\right)+S\left({\tilde{\omega }}_{i}\right)S\left({\tilde{\omega }}_{i}\right)$

$A_{i22}=S\left({\tilde{p}}_{i}^{*}\right)\left[S\left({\tilde{\varepsilon }}_{i}\right)+S\left({\tilde{\omega }}_{i}\right)S\left({\tilde{\omega }}_{i}\right)\right]-S\left({\tilde{a}}_{i}\right)$

$A_{i23}=S\left({\tilde{p}}_{i}^{*}\right){\tilde{a}}_{i}$

From 11 and 12, we have:

$$\begin{array}{c} {\tilde{f}}_{i}={\tilde{F}}_{i}+\sum_{j=i+1}^{n}{}_{j}^{i}R{\tilde{F}}_{j} \end{array}$$ (17)

$$\begin{array}{c} \begin{array}{cc} {\tilde{n}}_{i}= & {\tilde{N}}_{i}^{*}+\sum_{j=i+1}^{n}{}_{j}^{i}R{\tilde{N}}_{j}^{*}+{\tilde{p}}_{i}^{*}\times \left(\sum_{j=i+1}^{n}{}_{j}^{i}R{\tilde{F}}_{j}\right)\\ & +\left({}_{i+1}^{i}R{\tilde{p}}_{j+1}^{*}\right)\times \left(\sum_{j=i+2}^{n}{}_{j}^{i+1}R{\tilde{F}}_{j}\right)+\cdots \\ & +\left({}_{n-1}^{i}R{\tilde{p}}_{n-1}^{*}\right)\times {}_{n}^{i}R{\tilde{F}}_{n}\\ = & {\tilde{N}}_{i}^{*}+\sum_{j=i+1}^{n}{}_{j}^{i}R{\tilde{N}}_{j}^{*}+\sum_{k=i}^{n-1}\sum_{j=k+1}^{n}\left({}_{k}^{i}R{\tilde{p}}_{k}^{*}\right){\tilde{p}}_{i}^{*}\times {}_{j}^{i}R{\tilde{F}}_{j}\\ = & {\tilde{N}}_{i}^{*}+\sum_{j=i+1}^{n}{}_{j}^{i}R{\tilde{N}}_{j}^{*}+\sum_{j=i+1}^{n}\sum_{k=1}^{n}\left({}_{k}^{i}R{\tilde{p}}_{k}^{*}\right){\tilde{p}}_{i}^{*}\times {}_{j}^{i}R{\tilde{F}}_{j}\\ = & {\tilde{N}}_{i}^{*}+\sum_{j=i+1}^{n}({}_{j}^{i}R{\tilde{N}}_{j}^{*}+\sum_{k=1}^{n}\left({}_{k}^{i}R{\tilde{p}}_{k}^{*}\right){\tilde{p}}_{i}^{*}\times {}_{j}^{i}R{\tilde{F}}_{j}) \end{array} \end{array}$$ (18)

17 and 18 indicate that ${\tilde{f}}_{i}$ and ${\tilde{n}}_{i}$ can be expressed as

$$\begin{array}{c} {\tilde{f}}_{i}={\tilde{F}}_{i}+\sum_{j=i+1}^{n}{\tilde{f}}_{ij} \end{array}$$ (19)

$$\begin{array}{c} {\tilde{n}}_{i}=N_{i}^{*}+\sum_{j=i+1}^{n}{\tilde{n}}_{ij} \end{array}$$ (20)

In the 19, ${\tilde{f}}_{ij}={}_{j}^{i}R{\tilde{F}}_{j}$ reflects the influence of the dynamic properties of the link *j* on ${\tilde{f}}_{i}$, and ${\tilde{n}}_{ij}=({}_{j}^{i}R{\tilde{N}}_{j}^{*}+\sum_{k=1}^{n}\left({}_{k}^{i}R{\tilde{p}}_{k}^{*}\right){\tilde{p}}_{i}^{*}\times {}_{j}^{i}R{\tilde{F}}_{j})$ reflects the influence of the dynamic properties on ${\tilde{n}}_{i}$. We define:

$W_{i}=[\begin{array}{c} {\tilde{f}}_{i}\\ {\tilde{n}}_{i} \end{array}]$ , $W_{ij}=[\begin{array}{c} {\tilde{f}}_{ij}\\ {\tilde{n}}_{ij} \end{array}]$, $W_{i}^{*}=[\begin{array}{c} {\tilde{F}}_{i}\\ N_{i}^{*} \end{array}]$.

From 19 and 20, we have:

$$\begin{array}{c} W_{i}=W_{i}^{*}+\sum_{j=i+1}^{n}W_{ij} \end{array}$$ (21)

$$\begin{array}{c} \begin{array}{cc} W_{i,i+1}= & \left[\begin{array}{c} {\tilde{f}}_{i,i+1}\\ {\tilde{n}}_{i,i+1} \end{array}]=[\begin{array}{c} {}_{i+1}^{i}R{\tilde{F}}_{i+1}\\ {}_{i+1}^{i}R{\tilde{N}}_{i+1}^{*}+S\left({\tilde{p}}_{i}^{*}\right){}_{i+1}^{i}R{\tilde{F}}_{i+1} \end{array}\right]\\ = & \left[\begin{array}{cc} {}_{i+1}^{i}R & 0\\ S\left({\tilde{p}}_{i}^{*}\right){}_{i+1}^{i}R & {}_{i+1}^{i}R \end{array}][\begin{array}{c} {\tilde{F}}_{i+1}\\ {\tilde{N}}_{i+1}^{*} \end{array}\right]\\ & =T_{i}W_{i+1}^{*} \end{array} \end{array}$$ (22)

More generally, from 22, we have

$$\begin{array}{c} \begin{array}{cc} W_{ij}= & \left[\begin{array}{c} {\tilde{f}}_{ij}\\ {\tilde{n}}_{ij} \end{array}\right]\\ = & \left[\begin{array}{c} {}_{j}^{i}R{\tilde{F}}_{i+1}\\ {}_{j}^{i}R{\tilde{N}}_{j}^{*}+\sum_{k=i}^{j-1}{}_{k}^{i}RS\left({\tilde{p}}_{k}^{*}\right){}_{j}^{k}R{\tilde{F}}_{j} \end{array}\right]\\ = & T_{i}T_{i+1}\cdots T_{j-1}W_{j}^{*} \end{array} \end{array}$$ (23)

It can be concluded that

$$\begin{array}{c} W_{i}=A_{i}p^{i}+\sum_{j=i+1}^{n}T_{i}T_{i+1}\cdots T_{j-1}A_{j}p^{j}=\sum_{j=i}^{n}U_{ij}p^{j} \end{array}$$ (24)

where

$$U_{ij}=\{\begin{array}{c} A_{j}\left(i=j\right)\\ T_{i}T_{i+1}\cdots T_{j-1}A_{j}\left(i\neq j\right) \end{array}$$

Subsequently, we have:

$$\begin{array}{c} \begin{array}{cc} \tau_{i}= & \left[\begin{array}{cccccc} 0 & 0 & 0 & 0 & 0 & 1 \end{array}][\begin{array}{c} {}_{i}^{i-1}R{\tilde{f}}_{i}\\ {}_{i}^{i-1}R{\tilde{n}}_{i} \end{array}\right]\\ = & [\begin{array}{cccccc} 0 & 0 & 0 & 0 & 0 & 1 \end{array}][\begin{array}{cc} {}_{i}^{i-1}R & 0\\ 0 & {}_{i}^{i-1}R \end{array}][\begin{array}{c} {\tilde{f}}_{i}\\ {\tilde{n}}_{i} \end{array}]W_{i}\\ = & \sum_{j=i}^{n}Y_{ij}p^{i} \end{array} \end{array}$$ (25)

where

$$Y_{ij}=[\begin{array}{cccccc} 0 & 0 & 0 & 0 & 0 & 1 \end{array}][\begin{array}{cc} {}_{i}^{i-1}R & 0\\ 0 & {}_{i}^{i-1}R \end{array}]U_{ij}$$

Therefore, we have:

$$\begin{array}{c} \tau =[\begin{array}{c} \tau_{1}\\ \cdots \\ \tau_{n} \end{array}]=[\begin{array}{cccc} Y_{11} & Y_{12} & \cdots & Y_{1n}\\ 0 & Y_{22} & \cdots & Y_{2n}\\ 0 & 0 & \cdots & \cdots \\ 0 & 0 & 0 & Y_{nn} \end{array}][\begin{array}{c} p^{1}\\ p^{2}\\ \cdots \\ p^{n} \end{array}] \end{array}$$ (26)

By comparing 14 and 26, we can write the following matrix:

$$\begin{array}{c} Y=[\begin{array}{cccc} Y_{11} & Y_{12} & \cdots & Y_{1n}\\ 0 & Y_{22} & \cdots & Y_{2n}\\ 0 & 0 & \cdots & \cdots \\ 0 & 0 & 0 & Y_{nn} \end{array}] \end{array}$$ (27)

#### 3.2.2 Minimum parameter set

The dynamic parameters of each link can be expressed in the form of the following vector:

$$\begin{array}{c} X^{j}=[\begin{array}{cccccccccc} {XX}_{j} & {XY}_{j} & {XZ}_{j} & {YY}_{j} & {YZ}_{j} & {ZZ}_{j} & {MX}_{j} & {MY}_{j} & {MZ}_{j} & M_{j} \end{array}] \end{array}$$ (28)

Only the coupling relationship between the dynamic parameters is considered, so there are only 10 parameters for each link. Two methods are mainly used to reduce the number of parameters to be identified, i.e., to obtain the minimum parameter set: *QR* and *SVD*. If a column in the matrix *W* is always zero, the parameters corresponding to that column have no contribution to the model. To determine the columns that are always zero, we can substitute the several sets of data to calculate the matrix *W*, and we can easily determine the columns whose elements are always zero.

If the robot has *n* joints, a total of 10*n* parameters are included in *X*. We suppose that *c* parameters remain after removing some of the parameters that have no contribution to the model and take *r* groups of data to calculate the matrix *W*. Moreover, we assume *b* parameters in the minimum parameter set of the model, namely *rank*(*W*) = *b*. When *W* is less than the rank (*b* < *c*), the decomposition *QR* of *W* is not unique. However, if after the matrix *W* multiplied by a column permutation matrix *H*, *QR* decomposition of *WH* is the only:

$$\begin{array}{c} Q^{T}\left(WH\right)=[\begin{array}{c} T\\ 0_{(r-c)\times c} \end{array}]=[\begin{array}{cc} T_{1} & T_{2}\\ 0_{(r-b)\times b} & 0_{\left(r-b)\times (c-b\right)} \end{array}] \end{array}$$ (29)

where *Q* is still a positive definite matrix, *T*2 is a *b* × (*c* − *b*) matrix, and *T*1 is an upper triangular matrix.

In the above, the column replacement matrix *H* is obtained using *QR* decomposition, and the matrix *W* after column replacement is divided into two parts, as follows:

$$\begin{array}{c} WH=[\begin{array}{cc} W_{1} & W_{2} \end{array}] \end{array}$$ (30)

where *W*_1_ represents *b* independent columns, and *W*_2_ represents *c* − *b* columns that need to be eliminated (linearly dependent on the other columns). After the column transformation of *W*, the corresponding parameter order in *X* becomes:

$$\begin{array}{c} H^{T}X=[\begin{array}{c} {XB}_{1}\\ {XB}_{2} \end{array}] \end{array}$$ (31)

Let *XB*_2_ = 0, then

$$\begin{array}{c} \tau =WX=WHH^{T}X=[\begin{array}{cc} W_{1} & W_{2} \end{array}][\begin{array}{c} {XB}_{1}\\ 0 \end{array}]=W_{1}{XB}_{1} \end{array}$$ (32)

By applying the least-squares method to the above formula, we can obtain:

$$\begin{array}{c} {XB}_{1}={\left(W_{1}^{T}W_{1}\right)}^{-1}W_{1}^{T}\tau \end{array}$$ (33)

From 31, the following equation can be obtained:

$$\begin{array}{c} XB=H^{T}*[\begin{array}{c} {XB}_{1}\\ 0 \end{array}] \end{array}$$ (34)

## 4 Trajectory scaling and adaptive control algorithm design

The trajectory scaling method outputs a new desired trajectory, so it needs to be used in conjunction with the position controller. The force position control of the robot can be realized by combining the trajectory scaling method with the computed-torque control.

### 4.1 Trajectory scaling

In this section, the trajectory scaling method [31] is combined with the computed-torque controller to realize the force/position control of the robot. The idea of the trajectory scaling is to control the timestamp during the trajectory generation process by observing the difference between the actual joint torque of the robot and the expected joint torque, so as to change the expected trajectory and indirectly realize the force control. The implementation scheme is as follows: 1) First, based on the desired joint position, velocity, acceleration, and expected contact force, the desired joint torque is calculated using the inverse dynamic model; 2) Subsequently, through the transpose of the Jacobian matrix, the contact force is converted to the current torque of the joint, i.e., the measured torque of the joint; 3) The torque is measured minus the expected shutdown torque to obtain the torque error, whereby the timestamp in the trajectory generation process is varied to change the expected trajectory, thus indirectly realizing the control of the expected contact force. The trajectory scaling method outputs the new desired trajectory, so it needs to be used in conjunction with the position controller to achieve trajectory tracking and force control.

The expected trajectory is typically a function of time. The trajectory in the joint space can be expressed as *q*_*d*_(*t*) ∈ ^*n*^. For each time step *t*_*i*_, the calculation method of the original timestamp is:

$$\begin{array}{c} t_{i}=t_{i-1}+\Delta t \end{array}$$ (35)

where *t*_*i*−1_ represents the timestamp of the previous time step; Δ*t* is the time stepping value. By introducing the scaling factor $f_{s}(\Psi (\hat{r}))\in \left[-1,1\right]$, the time stepping value is associated with the expected trajectory and the actual contact force, where ${\hat{r}}_{i}$ represents the observed value of the external torque, which can be calculated through the expected trajectory and the measured joint torque:

$$\begin{array}{c} {\hat{r}}_{i}=\tau_{mea}-\hat{\tau } \end{array}$$ (36)

where *τ*_*mea*_ represents the measured value of the joint torque obtained from the conversion of the contact force at the end of the robot through the transpose of the Jacobian matrix; $\hat{\tau }$ represents the desired joint torque value corresponding to the expected trajectory of the previous time step, and the dynamic model of the mechanical arm can be used to calculate the following:

$$\begin{array}{c} \hat{\tau }=D\left(q_{d}\right){\ddot{q}}_{d}+C\left(q_{d},{\dot{q}}_{d}\right)+G\left(q_{d}\right) \end{array}$$ (37)

Therefore, the scaling factor *f*_*s*_ is a function of the expected trajectory $\left(q_{d},{\dot{q}}_{d},{\ddot{q}}_{d}\right)$ and the measuring joint torque *τ*_*mea*_, so the new time step can be written as:

$$\begin{array}{c} t_{i}=t_{i-1}+f_{s}(\Psi ({\hat{r}}_{i}))\Delta t \end{array}$$ (38)

where $\Psi ({\hat{r}}_{i})$ is defined as

$$\begin{array}{c} \Psi ({\hat{r}}_{i})=\frac{1}{\alpha_{\hat{r}}}(\frac{{\hat{r}}_{i}}{\tau_{\text{max}}}\cdot \frac{\Delta q_{d}^{i}}{\left\|\Delta q_{d}^{i}\right\|}) \end{array}$$ (39)

where $\Delta q_{d}^{i}=q_{d}^{i+1}-q_{d}^{i}$ represents the difference vector of two continuous expected joint positions; *τ*_*max*_ is a constant vector and represent the maximum torque of the joint; $\alpha_{\hat{r}}$ is the sensitivity of the trajectory scaling.

Therefore, the complete form of the scaling factor $f_{s}(\Psi (\hat{r}))$ is:

$$\begin{array}{c} f_{s}\left(\Psi \right)=\{\begin{array}{cc} \Phi (\Psi ) & 0\leq \Psi <1\\ 0 & 1\leq \Psi \leq 1+\Gamma \\ k\Phi (\Psi -(1+\Gamma ))-k & 1+\Gamma <\Psi \leq 2+\Gamma \\ -k & 2+\Gamma <\Psi \end{array} \end{array}$$ (40)

where *k* is a positive factor used to control the rate of decrease in the velocity, and is an optional dead zone. Here, the function Φ() ∈ [0, 1] is a monotone decreasing function, which is defined as:

$$\begin{array}{c} \Phi \left(\Psi \right)=\frac{1}{2}\left(1+\text{cos}\;\left(\pi \Psi \right)\right) \end{array}$$ (41)

Through the design of the above functions, the scaling factor *f*_*s*_ will control the expected position and velocity of the robot. When the measured torque is higher than the expected moment, the scaling factor *f*_*s*_ will decrease, making the robot to move slowly or in reverse motion until the desired moment is reached. The impact of the scaling factor *f*_*s*_ on the motion of the robot can be characterized as follows:

1. When *f*_*s*_ > 0, the robot moves forward (*f*_*s*_ = 1 is consistent with the expected trajectory);
2. When *f*_*s*_ = 0, the robot stops moving;
3. When *f*_*s*_ < 0, the robot moves in reverse.

### 4.2 Adaptive control algorithm

Considering the nonlinear and strong coupling characteristics of robot dynamics, the adaptive control method based on the computed torque has been widely studied, with significant achievements made thus far. Because of the huge amount of computation, it is difficult to apply the dynamics formula based on the Lagrangian method to the control of multi-degree-of-freedom robots. The recursive formula based on the Newton–Euler method is typically used to derive the dynamics equation, then obtain the regression matrix, and construct a control law through the adaptive estimation of the inertial parameters. The algorithm can effectively reduce the complexity of the control law and has little relationship with the degree of freedom of the robot.

The dynamic equations of the robot are described in 2 and 3. The variables *y* and *q*_*r*_ are respectively introduced and defined as follows:

$$\begin{array}{c} \begin{array}{c} e=q-q_{d}\\ \dot{e}=\dot{q}-{\dot{q}}_{d}\\ y=\dot{e}+\gamma e\\ {\dot{q}}_{r}={\dot{q}}_{d}-\gamma e \end{array} \end{array}$$ (42)

where *γ* ≥ 0, and we can deduce the following formula:

$$\begin{array}{c} y=\dot{q}-{\dot{q}}_{r} \end{array}$$ (43)

According to 2 and 3, one can obtain

$$\begin{array}{c} D\left(q\right)\ddot{q}-\dot{y}-C\left(q,\dot{q}\right)\left(\dot{q}-y\right)+G\left(q\right)=Y\left(q,\dot{q},{\dot{q}}_{r},{\ddot{q}}_{r}\right)P \end{array}$$ (44)

Subsequently:

$$\begin{array}{c} D\left(q\right)\dot{y}+C\left(q,\dot{q}\right)y=\tau -Y\left(q,\dot{q},{\dot{q}}_{r},{\ddot{q}}_{r}\right)P \end{array}$$ (45)

The following controller and adaptive law are designed to ensure the global stability of the system:

$$\begin{array}{c} \begin{array}{c} \tau =-K_{p}y+Y\left(q,\dot{q},{\dot{q}}_{r},{\ddot{q}}_{r}\right)\hat{P}\\ \dot{\hat{P}}=-\lambda Y^{T}\left(q,\dot{q},{\dot{q}}_{r},{\ddot{q}}_{r}\right)y \end{array} \end{array}$$ (46)

The stability proof of the control law is given below. We define $\tilde{P}=\hat{P}-P$, and then the Lyapunov function can be written as:

$$\begin{array}{c} V=\frac{1}{2}y^{T}D\left(q\right)y+\frac{1}{2}\lambda^{-1}{\tilde{P}}^{T}\tilde{P} \end{array}$$ (47)

By differentiating 47 with respect to time, we can obtain:

$$\begin{array}{c} \dot{V}=y^{T}D\left(q\right)\dot{y}+\frac{1}{2}y^{T}\dot{D}\left(q\right)y+\lambda^{-1}{\tilde{P}}^{T}\dot{\hat{P}} \end{array}$$ (48)

Based on the linearity and anti-symmetry of the robot dynamics equation, we arrive at:

$$\begin{array}{c} \begin{array}{cc} \dot{V}= & y^{T}D\left(q\right)\dot{y}+\frac{1}{2}y^{T}\dot{D}\left(q\right)y+\lambda^{-1}{\tilde{P}}^{T}\dot{\hat{P}}\\ = & y^{T}\left(\tau -YP-Cy\right)+\frac{1}{2}y^{T}\dot{D}\left(q\right)y+\lambda^{-1}{\tilde{P}}^{T}\dot{\hat{P}}\\ = & y^{T}\left(-K_{p}y+Y\hat{P}-YP\right)+\frac{1}{2}y^{T}\left(\dot{D}\left(q\right)-2C\right)y-{\tilde{P}}^{T}Y^{T}y\\ = & y^{T}\left(-K_{p}y+Y\tilde{P}\right)-{\tilde{P}}^{T}Y^{T}y\\ = & -y^{T}K_{p}y\\ \leq & 0 \end{array} \end{array}$$ (49)

Hence, it is proven that the robot is stable when using the control law.

### 4.3 Finding the regression matrix

From 17–28, we find that the regression matrix *Y* can be expressed as a function of ${\tilde{\omega }}_{i}$, ${\tilde{\varepsilon }}_{i}$, and ${\tilde{v}}_{i}$. Therefore, the expressions of ${\tilde{\omega }}_{ir}$, ${\tilde{\varepsilon }}_{ir}$, and ${\tilde{v}}_{ir}$ for obtaining $Y(q,\dot{q},{\dot{q}}_{r},{\ddot{q}}_{r})$ are given as follows:

$$\begin{array}{c} {\tilde{\omega }}_{ir}={}_{i-1}^{i}R\left({\tilde{\omega }}_{i-1}+z{\dot{q}}_{ir}\right)\left({\tilde{\omega }}_{0}=0\right) \end{array}$$ (50)

$$\begin{array}{c} {\tilde{\varepsilon }}_{ir}={}_{i-1}^{i}R[{\tilde{\varepsilon }}_{i-1}+{\tilde{\omega }}_{i-1}\times z{\dot{q}}_{ir}+z{\ddot{q}}_{ir}]\left({\tilde{\varepsilon }}_{0}=0\right) \end{array}$$ (51)

$$\begin{array}{c} {\tilde{v}}_{ir}={}_{i-1}^{i}R\left({\tilde{v}}_{i-1}+z{\dot{q}}_{i}\right)+{\tilde{\omega }}_{i}\times {\tilde{p}}_{i}^{*} \end{array}$$ (52)

Thus, we have $Y(q,\dot{q},{\dot{q}}_{r},{\ddot{q}}_{r})$.

**Remark 1** *Using regression matrix can implement the known information in dynamics as much as possible, and take the identified parameters as the initial value of the adaptive controller. In this case, the calculated dynamic model is more accurate. Relatively speaking, the adaptive control method does not require the initial value of dynamic parameters, so the dynamic model error is large when the robot is just running. It is worth mentioning that, another common scheme of robot adaptive control is to use fuzzy logic system and radial basis function neural network combined with adaptive estimator to deal with the uncertainty of dynamic model. In this case, most known information of dynamics can not be used; Because the initial value of the estimated parameter needs to be set according to experience, and it will bring large model error before approaching the true value. However, the control scheme proposed in this paper does not have this problem*.

## 5 Numerical simulation

To verify the feasibility of the proposed control method, MATLAB/Simulink is applied to build the robot platform with UR5 for real-time simulation. UR5 has six links and six joints, whose parameters are shown in Table 1. The simulation experiment in this chapter firstly verifies the validity of parameter identification algorithm and obtains the identification results of dynamic parameters. Then, these identification results are used as the initial state of the adaptive parameter estimates to verify the effectiveness of the control algorithm combining trajectory stretching and adaptive.

**Table 1:** Model parameters of the robot.

Link number	*d*(*m*)	*a*(*m*)	*α*(°)	Mass(kg)	Inertia matrix (*kg* ⋅ *m*^2^)
1	0	0.0892	90	3.7	diag([0.010267495893, 0.010267495893,0.00666])
2	0.425	0	0	9.393	diag([0.22689067591, 0.22689067591,0.0151074])
3	0.392	0	0	2.275	diag([0.049443313556, 0.049443313556,0.004095])
4	0	0.1093	90	1.219	diag([0.111172755531, 0.111172755531, 0.21942]
5	0	0.09475	-90	1.219	diag([0.111172755531, 0.111172755531, 0 0.21942]
6	0	0.0825	0	0.1897	diag([0.0171364731454, 0.0171364731454, .033822]

### 5.1 Verification of parameter identification method

**Fig 3:**
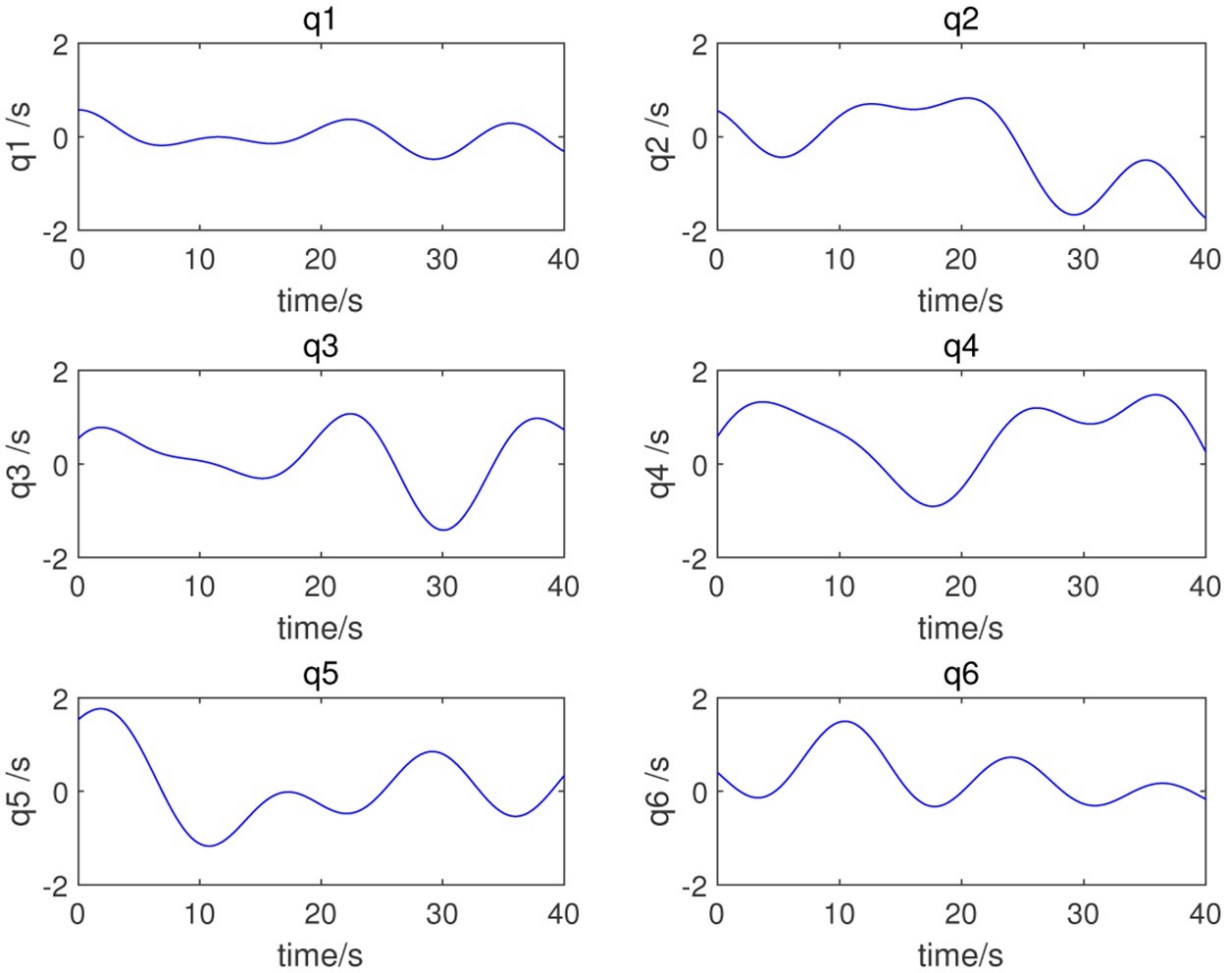
Excitation trajectory.

The aim of the excitation trajectory design is to excite the robot sufficiently so as to obtain the dynamic parameters of the robot more accurately. For a given robot, the optimal excitation trajectory is theoretically the one that minimizes the error of dynamic parameter identification using the excitation trajectory. The type of excitation trajectory is the finite-term Fourier-series trajectory, and the parameters of the excitation trajectory need to be optimized depending on the actual situation. Since the Fourier-series trajectory is used as the excitation trajectory, the Fourier-series trajectory of joint *i* is expressed as follows:

$$\begin{array}{c} \begin{array}{c} q_{i}\left(t\right)=q_{i0}+\sum_{k=1}^{5}a_{i,k}\;\text{sin}\;\left(k\omega_{f}t\right)+\sum_{k=1}^{5}b_{i,k}\;cos\;\left(k\omega_{f}t\right)\\ {\dot{q}}_{i}\left(t\right)={\dot{q}}_{i0}+\sum_{k=1}^{5}a_{i,k}k\omega_{f}\;cos\;\left(k\omega_{f}t\right)-\sum_{k=1}^{5}b_{i,k}k\omega_{f}\;sin\;\left(k\omega_{f}t\right)\\ {\ddot{q}}_{i}\left(t\right)={\dot{q}}_{i0}-\sum_{k=1}^{5}a_{i,k}k^{2}\omega_{f}^{2}\;sin\;\left(k\omega_{f}t\right)-\sum_{k=1}^{5}b_{i,k}k^{2}\omega_{f}^{2}\;cos\;\left(k\omega_{f}t\right) \end{array} \end{array}$$ (53)

where *q*_*i*_(*t*), ${\dot{q}}_{i}\left(t\right)$, and ${\ddot{q}}_{i}\left(t\right)$ are respectively the angle, angular velocity, and angular acceleration of joint *i* at time *t*. *ω*_*f*_ is the interval angular frequency, *k* is the number of harmonic terms of the Fourier series, *a*_*i,k*_ and *b*_*i,k*_ are the amplitudes of the sine and cosine terms of joint *i*. In this simulation, *ω*_*f*_ = 0.1*π*, and the coefficient of Fourier series is obtained by dynamic programming. Finally, the coefficient combinations, listed in Table 2, can be obtained; Fig 3 shows the excitation trajectories of each joint.

**Table 2:** Values of the dynamic parameters.

*q*_1,0_	-0.0620	*q*_2,0_	-0.2349	*q*_3,0_	0.1538	*q*_4,0_	0.4295	*q*_5,0_	0.1625	*q*_6,0_	0.1310
*a*_1,1_	0.2939	*a*_2,1_	0.4694	*a*_3,1_	-0.1874	*a*_4,1_	-0.0346	*a*_5,1_	-0.2425	*a*_6,1_	0.4603
*b*_1,1_	0.0283	*b*_2,1_	0.5000	*b*_3,1_	-0.0955	*b*_4,1_	-0.1512	*b*_5,1_	0.1959	*b*_6,1_	0.1731
*a*_1,2_	-0.2678	*a*_2,2_	-0.2170	*a*_3,2_	0.3604	*a*_4,2_	0.1765	*a*_5,2_	0.0285	*a*_6,2_	0.1446
*b*_1,2_	-0.1137	*b*_2,2_	-0.4998	*b*_3,2_	-0.5000	*b*_4,2_	-0.4807	*b*_5,2_	0.4858	*b*_6,2_	0.1124
*a*_1,3_	0.4999	*a*_2,3_	0.0069	*a*_3,3_	0.4925	*a*_4,3_	0.4607	*a*_5,3_	0.3191	*a*_6,3_	-0.0431
*b*_1,3_	-0.4838	*b*_2,3_	0.0907	*b*_3,3_	0.4999	*b*_4,3_	-0.4999	*b*_5,3_	0.4788	*b*_6,3_	0.0483
*a*_1,4_	0.4999	*a*_2,4_	0.3284	*a*_3,4_	0.4999	*a*_4,4_	0.4998	*a*_5,4_	-0.0705	*a*_6,4_	-0.3041
*b*_1,4_	-0.5000	*b*_2,4_	0.3611	*b*_3,4_	0.0102	*b*_4,4_	-0.1715	*b*_5,4_	0.3731	*b*_6,4_	-0.1899
*a*_1,5_	0.5000	*a*_2,5_	-0.4999	*a*_3,5_	-0.2774	*a*_4,5_	0.0455	*a*_5,5_	0.3600	*a*_6,5_	-0.3387
*b*_1,5_	0.5000	*b*_2,5_	0.3386	*b*_3,5_	0.4764	*b*_4,5_	0.4992	*b*_5,5_	-0.1627	*b*_6,5_	0.2237

The model of the robot, listed in Table 1, obtained using the Newton–Euler method contains 60 dynamic parameters. By using the dimensionality reduction method of the model proposed in this paper and grouping the dynamic parameters, we can obtain a set containing 51 dynamic parameters, including 36 fully identifiable parameters and 15 identified parameters after linear combination. The remaining nine are unidentifiable parameters.

**Fig 4:**
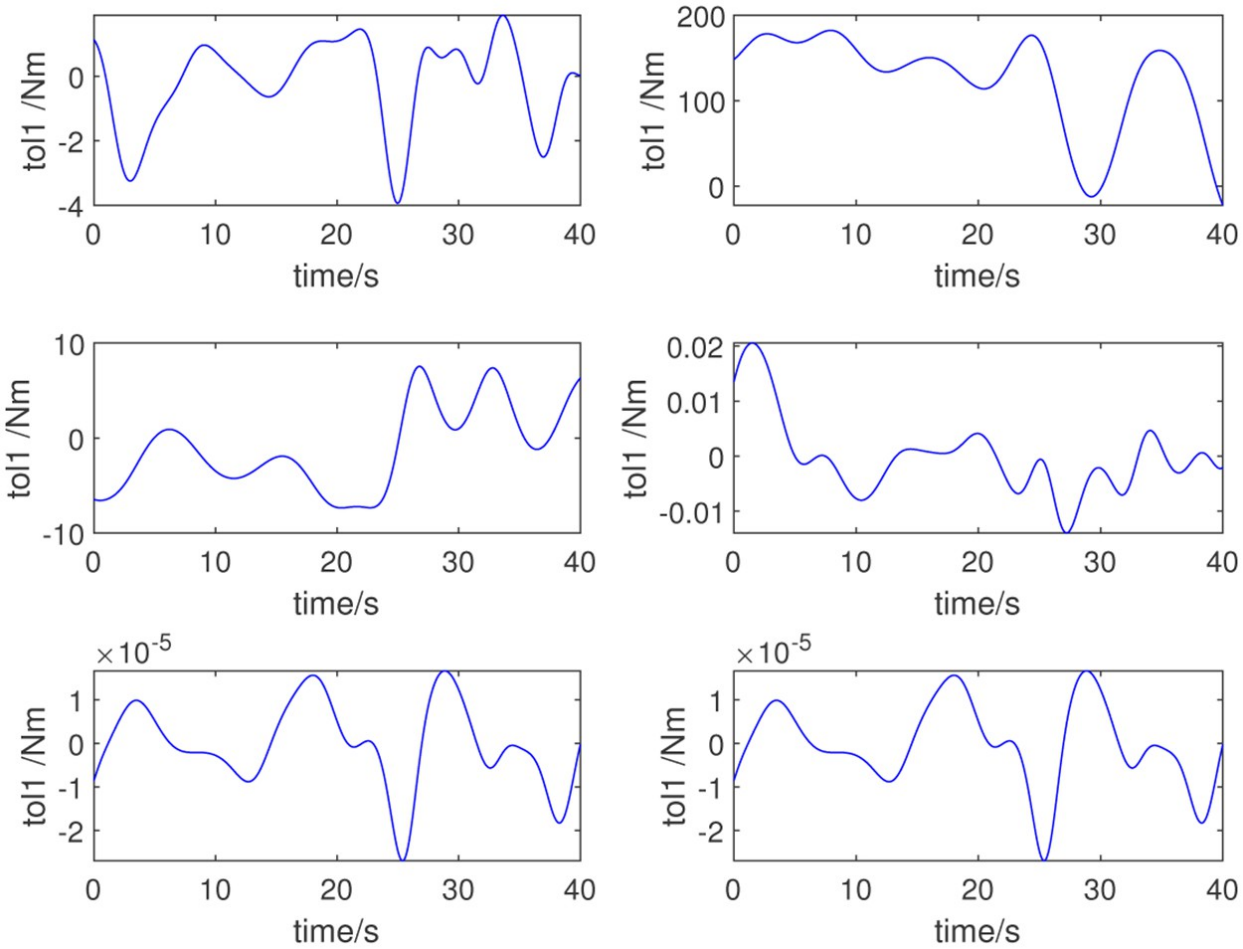
Joint torque.

**Fig 5:**
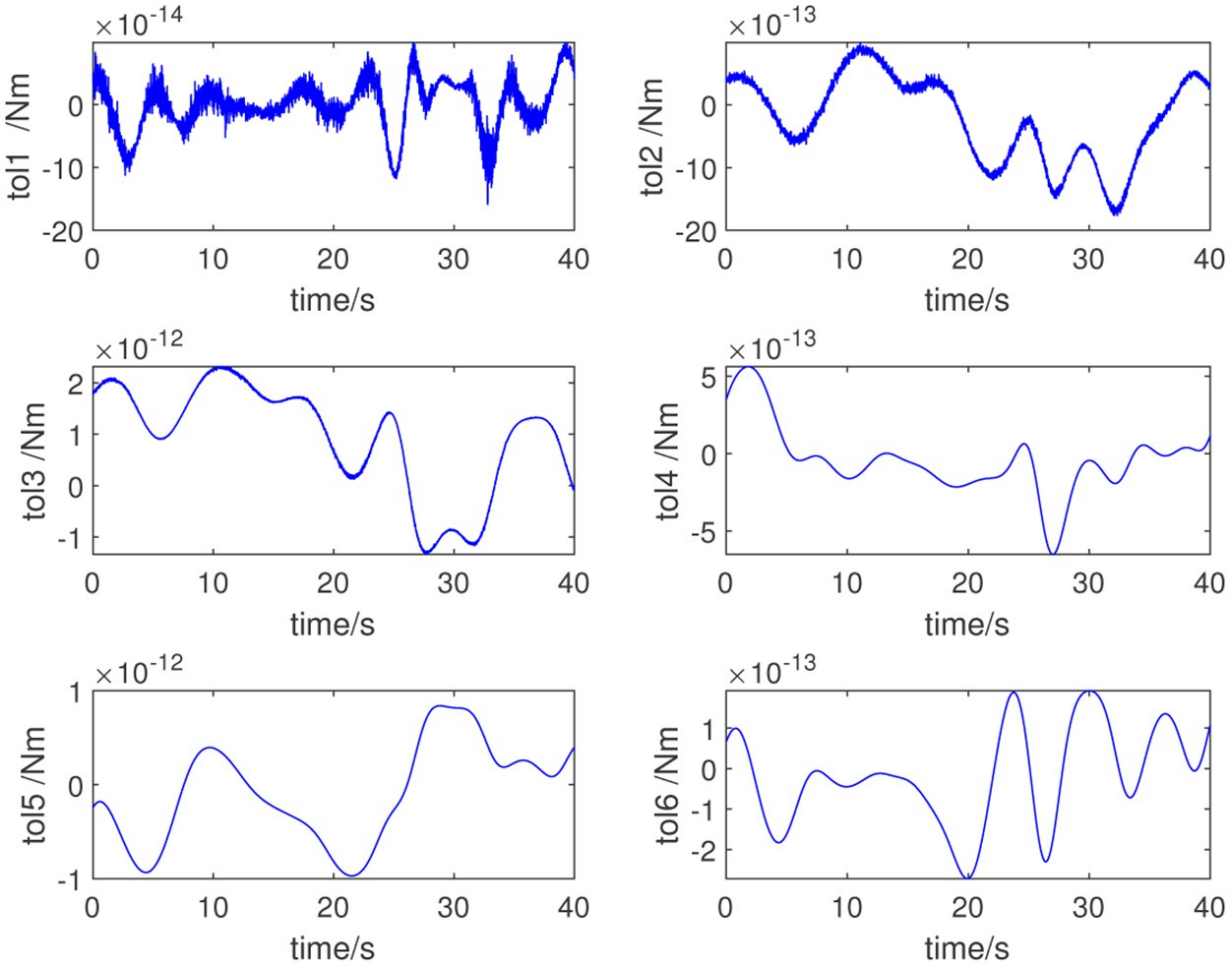
Errors in the measured and computed torques.

**Fig 6:**
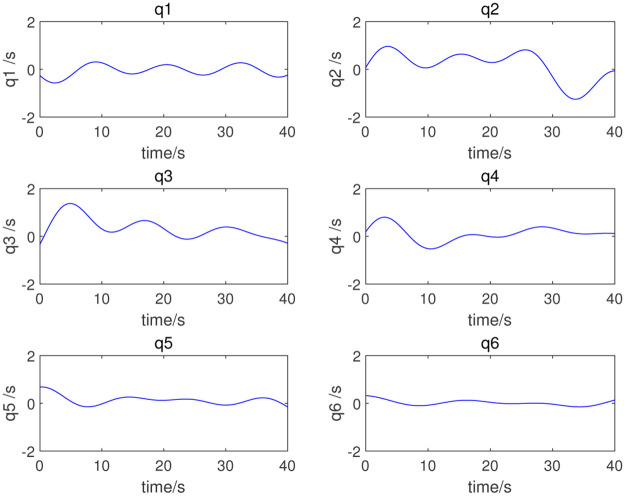
Excitation trajectory for further verification.

In this study, the dynamic parameters obtained via the parameter identification method are used for the adaptive control of the selected robot. We input the numerical value of the excitation trajectory to the robot, and then by measuring the joint angle, angular velocity, angular acceleration, and joint torque of the robot, we can complete the dynamic parameter identification. The identification method proposed in this paper is adopted; Table 3 lists the dynamic parameters obtained. Fig 4 shows the measured joint torque. Fig 5 shows the errors in the measured and computed torque. As shown in Figs 5 and 6, the measured and computed torques are largely the same, with an error of less than 10^−12^*Nm*. The simulation results show that the inertial parameters identified are effective for the excitation trajectory and joint torque.

**Table 3:** Coefficients of the excitation trajectory.

number	value	number	value	number	value	number	value	number	value
1	0	12	0	23	0	34	0	45	0.0002
2	0	13	0	24	0	35	0.0004	46	0
3	0.0389	14	0.0068	25	0.0016	36	0	47	0
4	0	15	-1.0451	26	0	37	0	48	0
5	0	16	0.0047	27	0	38	0	49	0
6	-0.0243	17	1.0943	28	0	39	0	50	0
7	0.1463	18	-0.4109	29	-5.6216	40	0.0029	51	0
8	-3.9301	19	0	30	0	41	0		
9	0.1068	20	-7.0953	31	20.873	42	0		
10	0.4289	21	0	32	0	43	0		
11	0	22	0.0003	33	0	44	0		

**Fig 7:**
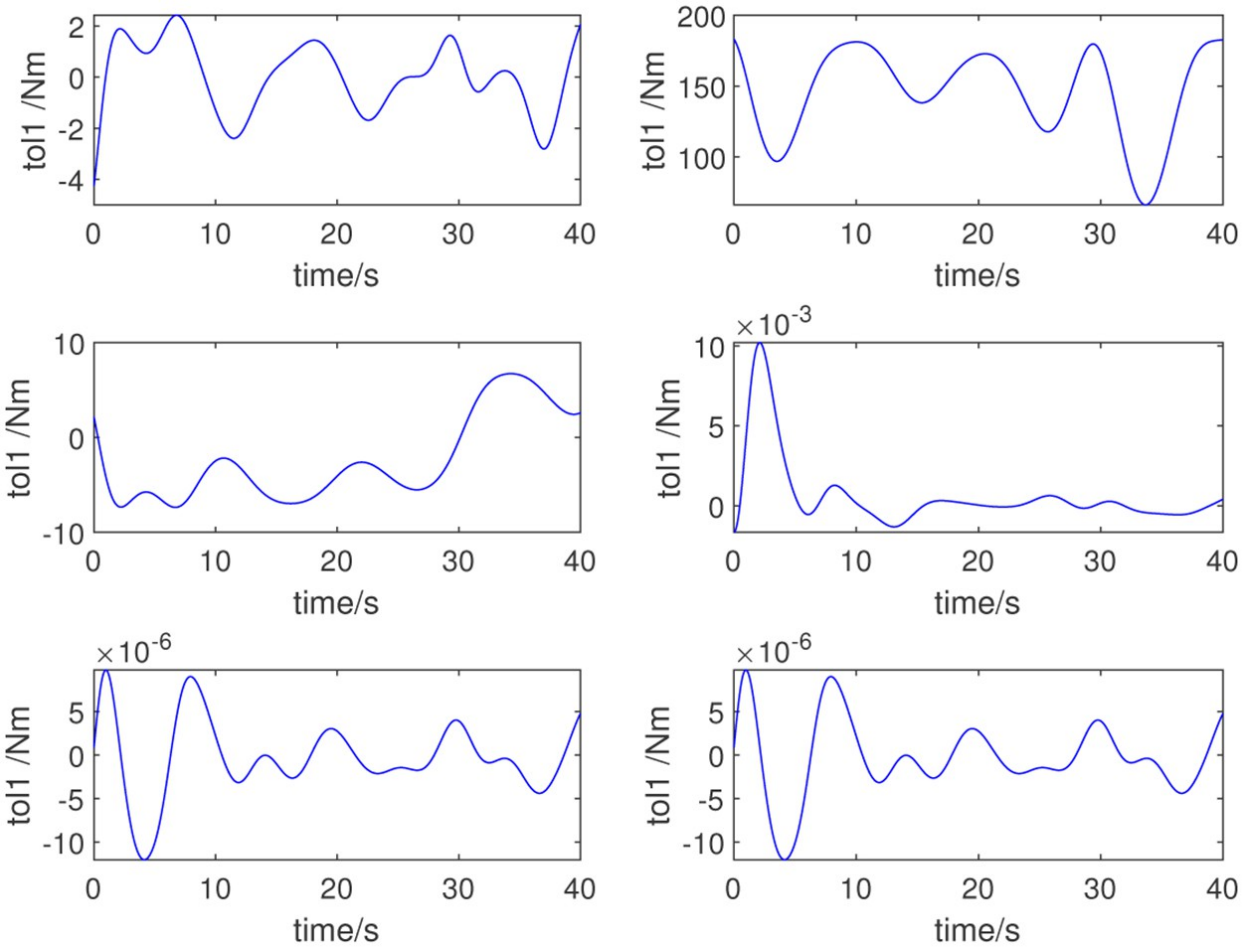
Joint torque for further verification.

**Fig 8:**
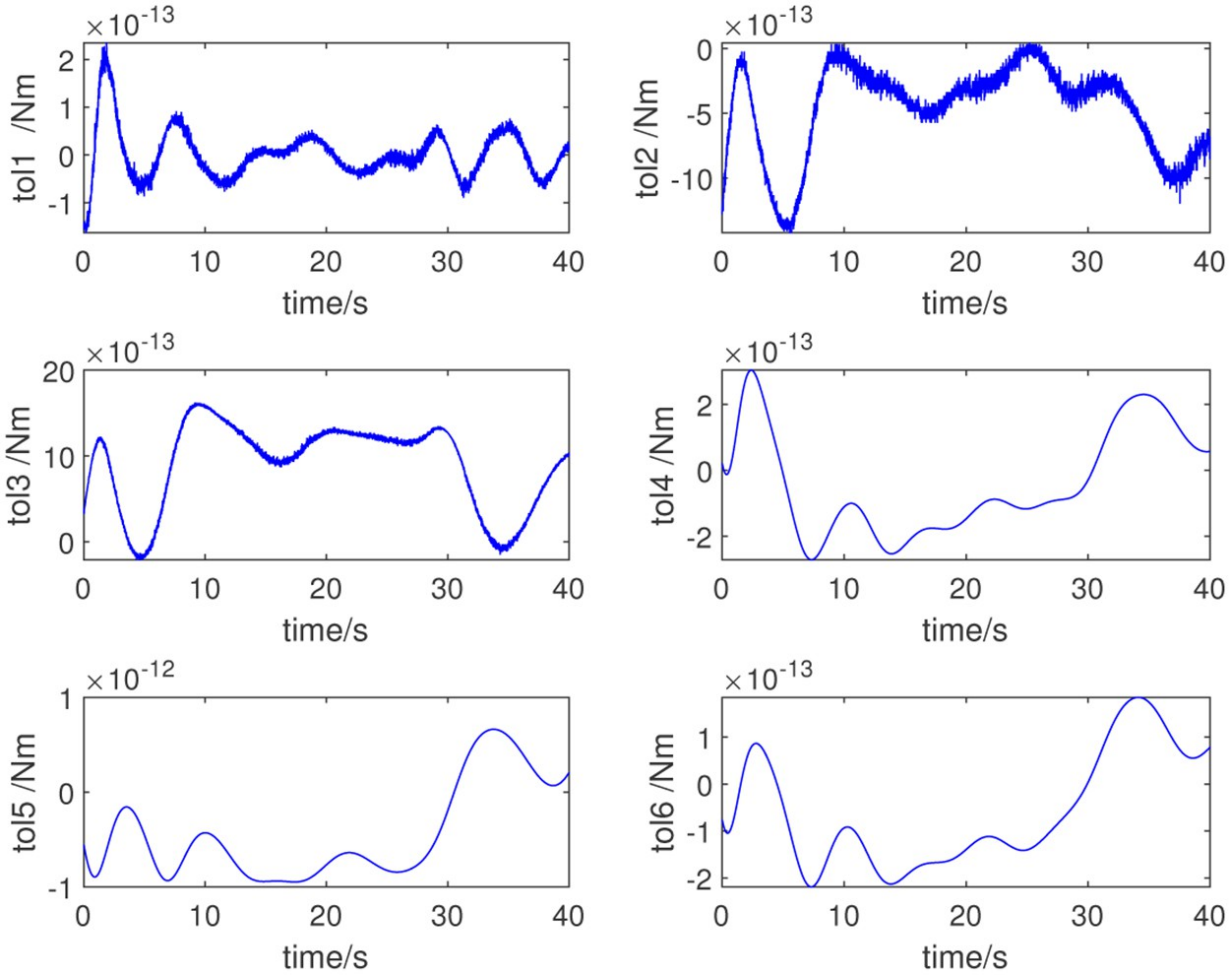
Errors in the measured and computed torques for further verification.

To further verify the validity of the identified inertial parameters, another set of excitation trajectories is used for verification. Fig 6 shows the new excitation trajectory. Fig 7 shows the measured torque under this set of trajectories; Fig 8 shows the error. The validation results confirm the validity of the dynamic parameter identification method and the results.

### 5.2 Verification of control algorithm

Based on the trajectory scaling and adaptive control algorithm, we carry out a simulation verification in this part. The same robot model is used for the dynamic parameter identification, as listed in Table 1.

**Fig 9:**
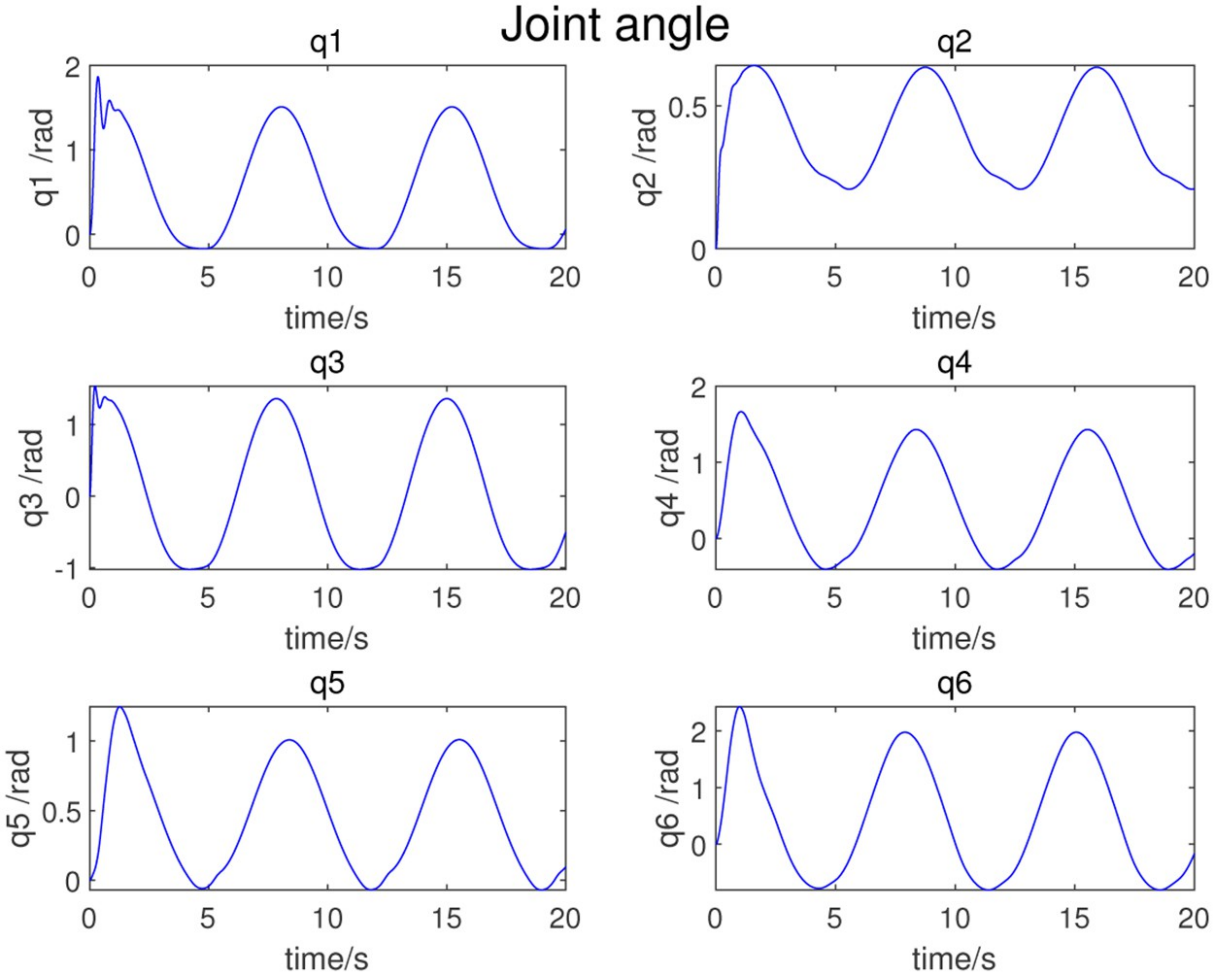
Trajectory of 1st joint.

**Fig 10:**
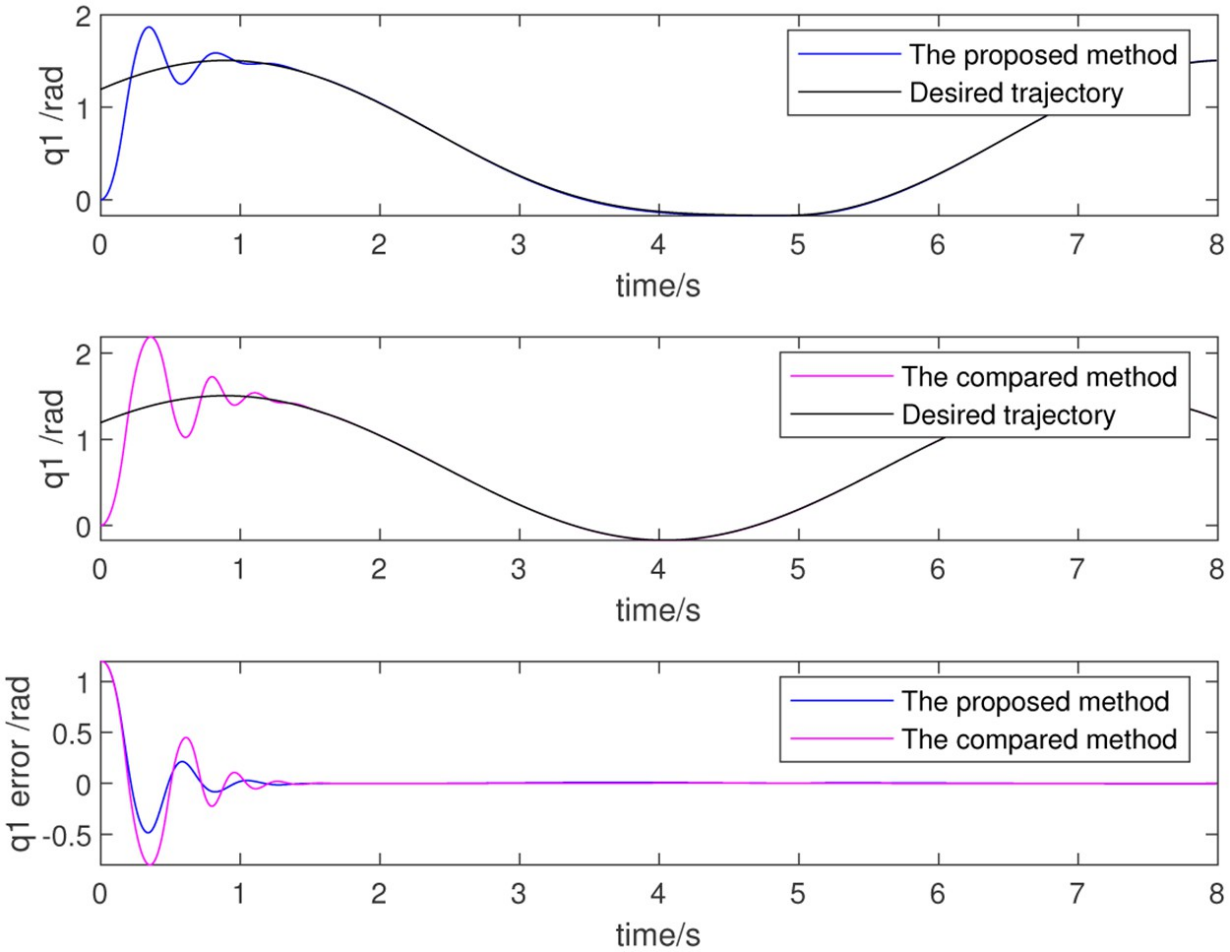
Trajectory of 2nd joint.

**Fig 11:**
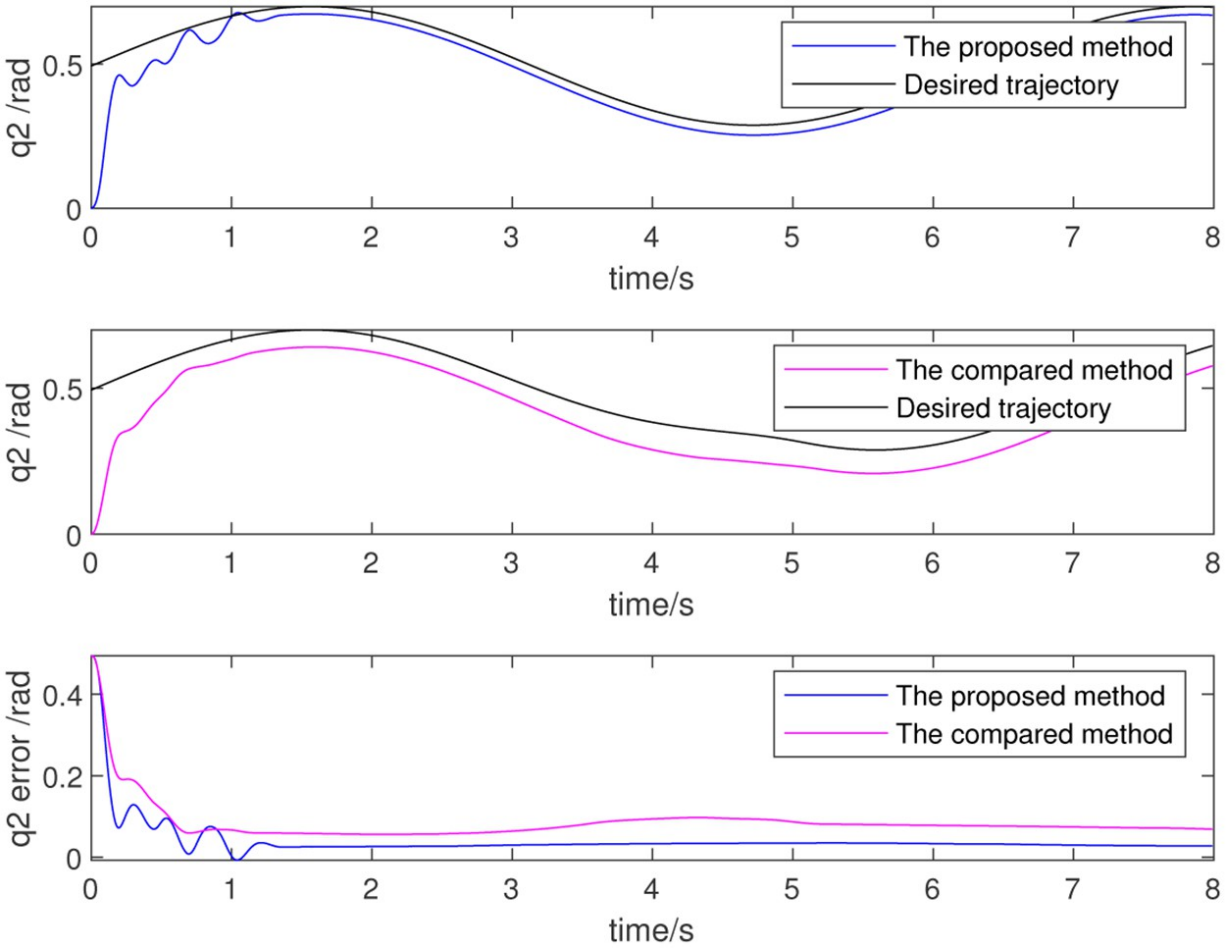
Trajectory of 3rd joint.

In the trajectory scaling module, the sensitivity of the scaling *α* = 10, the speed decreasing factor *k* = 1, and optional dead zone *Γ* = 0.5. In the adaptive torque control module,*γ* = 20*eyes*(6),*K*_*p*_ = *diag*([10,20,10,0.02,0.02,0.02]), and λ = 0.01 * *eye*(60). The simulation of a given input trajectory is conducted with the following parameters: *q*_*d*_ = *q*_*d*0_ + *Amp* * sin(*ω*_0_ *t*) + *Bmp* * *cos*(*ω*_0_ *t*), including *q*_*d0*_ = [0.1, 0.2, 0.3, 0.1, 0.2, 0.3], *Amp* = [0.1, 0.2, 0.3, −0.1, −0.2, −0.3], *Bmp* = [−0.1, −0.2, −0.3, 0.1, 0.2, 0.3], *ω*_0_ = 4, and the initial values of the adaptive estimation parameters are 0. Figs 9–11 show the simulation results. Fig 9 shows that the motion trajectory of the joint can effectively track the given trajectory. Fig 10 shows the tracking error. Fig 11 shows each shutdown torque. The results show that the algorithm is effective.

**Fig 12:**
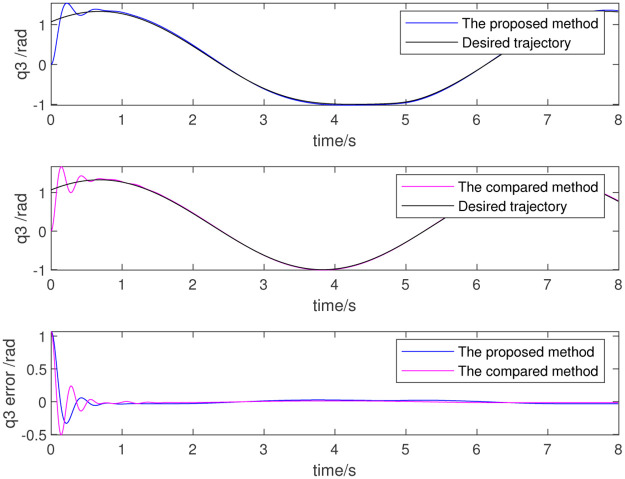
Trajectory of 4th joint.

**Fig 13:**
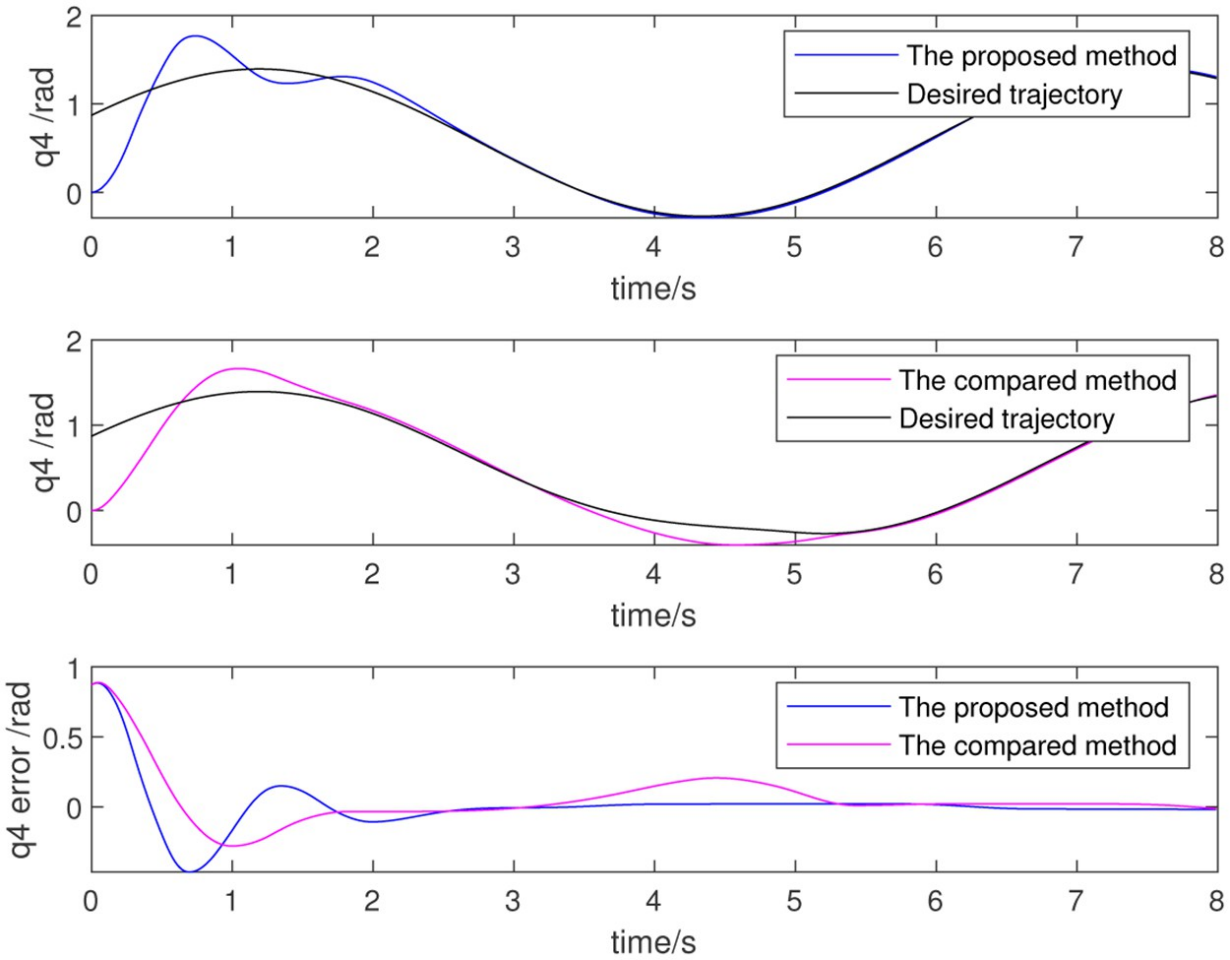
Trajectory of 5th joint.

**Fig 14:**
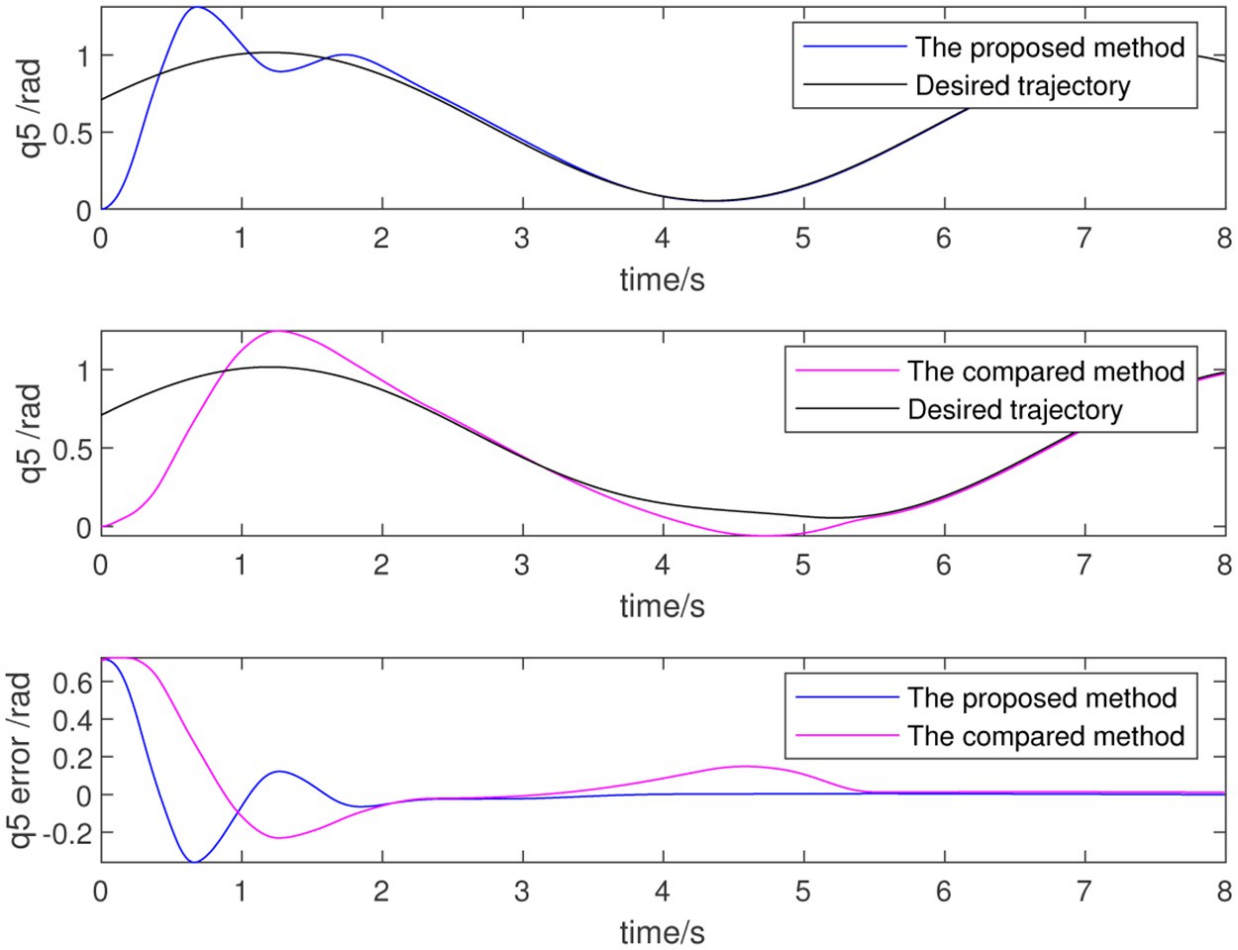
Trajectory of 6th joint.

To further verify the effect of dynamic parameter identification, the identified results are used as initial values for the adaptive parameter estimation as part of a re-simulation. Figs 12–14 show the results, which respectively represent the trajectory tracking results and joint torque. The control effect is evident because the initial value is non-zero. The above simulation results show that the trajectory tracking error of the robot is very small and that the control precision is very high when using data identified from the dynamic parameters. This also proves the effectiveness of the dynamic parameter identification method and the robot control method.

## 6 Conclusion

In this study, a robot dynamics model is established using the Newton–Euler method to determine the regression matrix and parameter vectors. The order of the regression matrix is reduced, and the minimum parameter set is obtained. The validity of the method is proven using the verification trajectory. We employe force control for the outer loop based on trajectory scaling and position control for the inner loop based on the adaptive torque method to achieve a high-precision control performance and compliance control of the robot. The proposed algorithm is verified by conducting a simulation. As part of future work, we plan to verify our approach on an experimental platform suitable for a real robot and further develop a compliant control algorithm for the force.
